# Monitoring the effects of oxidative stress on the growth of Holstein bull calves using Diquat

**DOI:** 10.3389/fvets.2025.1573555

**Published:** 2025-03-21

**Authors:** Ting Liu, Huan Chen, Dongzhu Cairang, Shuru Cheng, Zhihao Luo, Ming Zhang, David P. Casper

**Affiliations:** ^1^College of Animal Science and Technology, Gansu Agricultural University, Lanzhou, China; ^2^College of Veterinary Medicine, Gansu Agricultural University, Lanzhou, China; ^3^Gansu Devotion Biotech Co., Ltd., Lanzhou, China; ^4^Casper’s Calf Ranch, LLC, Freeport, IL, United States; ^5^Department of Animal Science, North Carolina A&T State University, Greensboro, NC, United States

**Keywords:** Diquat, oxidative stress, weaned calves, serum antioxidant, fecal score

## Abstract

**Background:**

Holstein bull calves received a one-time intraperitoneal injection of Diquat to explore its effects on growth, body frame, blood oxidation indices, fecal scores, and pathogenic bacteria in weaned calves.

**Methods:**

A total of twelve 70-day-old Holstein bull calves with similar body weight (BW) and body condition were randomly assigned to one of four treatments. The treatments were as follows: Control: calves were injected with 0 mg/kg BW Diquat in 0.9% sterilized saline; treatments 6, 8, and 10 mg/kg BW Diquat, respectively. The experimental period lasted for 24 days. Measurements of BW, average daily gain (ADG), fecal scores, frame gains, fecal pathogen count, and blood samples for monitoring oxidative stress were collected on days 0, 6, 12, 18, and 24. Data were analyzed using a randomized complete block design, with days considered as a repeated measurement. In addition, exponential polynomial contrasts were used to assess the linear, quadratic, and cubic treatment responses.

**Results:**

Growth performance (BW) and ADG showed a cubic response (*p* < 0.02), initially decreasing and then increasing with higher Diquat dosages. Fecal scores and fecal ratios exhibited a quadratic response (*p* < 0.02), rising at a diminishing rate as Diquat injection dosages increased. Frame gains for body slope, body length, hip height, and abdominal girth displayed a linear decrease (*p* < 0.03) with increasing Diquat injection dosages. Serum aspartate aminotransferase, glutathione, total antioxidant capacity, catalase, malondialdehyde, cortisol, and noradrenaline concentrations revealed a linear increase (*p* < 0.01) in response to higher Diquat injection dosages, while alanine transaminase, superoxide dismutase, and glutathione peroxidase demonstrated a quadratic response (*p* < 0.02), increasing at a diminishing rate. Fecal *Escherichia coli* concentrations demonstrated a cubic response (*p <* 0.01), while *Staphylococcus aureus* and *Salmonella-Shigella* demonstrated linear increases (*p* < 0.01) with increasing Diquat dosages.

**Conclusion:**

Diquat injection induced oxidative stress, leading to reduced growth performance, along with increased serum oxidative stress indices, fecal scores, and fecal pathogens, a response that may persist for up to 24 days. An optimal dosage of 8 mg/kg BW is proposed as a benchmark for elucidating oxidative stress to evaluate future technologies aimed at reducing, eliminating, or preventing oxidative stress.

## Introduction

Oxidative stress is defined as an excess (imbalance) of cellular concentrations of free radicals within the body, which prevents the calf from effectively utilizing antioxidants for metabolizing these highly reactive and unstable radicals, ultimately resulting in physiological dysfunction (1). The impact of systemic and local oxidative status on neonatal calf diarrhea remains unclear. External factors or physiological regulatory disorders can lead to the production of excessive quantities of oxygen free radicals that the calf cannot metabolize in a timely manner, resulting in intracellular reactive oxygen species (ROS) concentrations that exceed the calf’s antioxidant capacity. This imbalance in oxidation-antioxidant dynamics ultimately leads to inflammation and tissue damage (2).

Early weaning technologies have been widely adopted in intensive dairy farming operations. These practices can reduce calf-rearing costs, improve reproductive efficiency, and enhance economic profitability. However, early weaning may result in stressful situations that lead to increased blood cortisol levels, neutrophilia, decreased lymphocyte counts, and lowered immunity (3). Calves experience significant stress during weaning (4), which can negatively impact their health and growth performance (5). When calves undergo weaning stress, the balance between ROS production and the scavenging of ROS by endogenous defense molecules, such as superoxide dismutase (SOD), catalase (CAT), and glutathione peroxidase (GSH-Px), becomes disrupted (6). Elevated ROS production and insufficient scavenging can result in tissue damage and oxidative stress due to the oxidation of proteins, lipids, and nucleic acids (7, 8), ultimately leading to a reduction in feed intake, growth, and muscle development (9). The stress related to weaning also leads to villus atrophy and increased crypt depth, which compromises the intestinal barrier, resulting in inflammation, diarrhea, malabsorption, and systemic diseases. This ultimately affects the health and growth status of the animals (10, 11).

An oxidant can be used as a physiological stressor to induce oxidative stress in animals. Previous research has reported that an oxidant can be administered to induce oxidative stress, exceeding the animal’s physiological control mechanisms for managing oxidative stress, which affects growth performance and health along with its mechanisms of action (12–14). The three most common oxidants are hydrogen peroxide (H_2_O_2_), oxidized fish oil, and Diquat. The use of H_2_O_2_ results in irreversible cellular senescence, which is problematic, even though H_2_O_2_ is often used to model oxidative stress in animal tissues (15) or in cells (16). Oxidized fish oil can induce oxidative stress when fed to mice (17) and fish (18). Animals produce more oxidants than they can scavenge, resulting in an imbalance between their oxidative and antioxidant systems, which causes tissue damage and growth inhibition. The challenge of using oxidized fats and oils lies in controlling the complex production conditions and standardizing the oxidation products and concentrations, which limits their application for inducing oxidative stress in calves (19). In contrast to oxidized fish oil, the use of Diquat to induce oxidative stress can be easily managed through dosage concentration and the number of injections for standardization (18).

Diquat is a bipyridine herbicide that converts oxygen molecules into superoxide anion radicals and stimulates the production of cellular free radicals through cyclic reduction–oxidation processes (20). This cyclic reduction–oxidation process further promotes the generation of intracellular free radicals (20). Previous research has shown that Diquat injections induced oxidative stress in mice (21, 22), poultry (23, 24), piglets (25), and sheep (26), which resulted in reduced growth performance and nutrient metabolism (27). Lv et al. (25) reported that a 10 mg/ kg body weight (BW) Diquat injection significantly increased serum malondialdehyde (MDA) concentration (*p* < 0.05) while inhibiting the activities of superoxide dismutase (SOD) and glutathione peroxidase (GSH-Px), both of which are antioxidants, in piglets.

Fu et al. (1) reported that wild-type rats injected with Diquat at 12 mg/ kg BW intraperitoneally experienced oxidative stress without causing death. Zhao (26) reported that adult small-tailed cold (frigid) sheep injected intraperitoneally with Diquat at 12 mg/kg BW suffered from excessive oxidative stress and mortality, whereas chronic oxidative stress lasting 24 days was induced in adult Billy goats through the intraperitoneal injection of Diquat at 10 mg/kg BW. To the best of our knowledge, Diquat has not been reported in the literature regarding the use of Diquat as an oxidant to develop and monitor oxidative stress in calves.

Based on the aforementioned literature, particularly the studies conducted by Fu et al. (1) and Zhao (26), Diquat has been selected as the oxidant source to induce and monitor oxidative stress in Holstein calves at three specific dosages: 6, 8, and 10 mg/kg BW. In the present study, Diquat is utilized for the first time to induce oxidative stress in calves. The objective is to ascertain the optimal injected dosage of Diquat for elucidating oxidative stress while concurrently monitoring the severity and duration of this stress. This study tests the hypothesis that oxidant-induced oxidative stress, which exceeds physiological control mechanisms, can be effectively monitored in relation to its impact on the growth performance and health of Holstein calves, as well as the underlying mechanisms of action.

## Materials and methods

### Ethics statement

The animal study was approved by the Laboratory Animal Ethics Committee of Gansu Agricultural University (GSAU-ETH-AST-2023-036). The study was conducted in accordance with local legislation and institutional requirements.

### Calf selection and feeding

The calves were managed, cared for, and fed according to the guidelines outlined in the 4th edition of the “Guide for the Care and Use of Agricultural Animals in Research and Teaching,” published by ADSA-ASAS-PSA (29), to supplement the Chinese guidelines. This experiment was conducted at the Shengyuan Farming Base of the Liaoyuan Dairy Group in Linxia Hui Autonomous Prefecture, Gansu Province, China. The facility was naturally ventilated, with pens cleaned every day, disinfection performed every 3 days, and the straw bedding changed weekly. Calves always had access to fresh water on a free-choice basis.

Twelve Holstein bull calves, similar in body condition, birth dates, and BW (70 ± 3 days old, 87.99 ± 2.33 kg), were selected from the dairy herd and randomly assigned to one of four treatments using an RCBD (30) with a 24-day experimental period. The calves had previously been weaned at 56 days. Post-weaned calves were chosen because pre-weaned calves have a limited immune system (31), and weaning stress would not confound the Diquat results. The treatments were as follows: Control (0): calves were injected with 0 mg/kg BW Diquat in 20 mL of 0.9% sterile saline; 6 mg/ kg BW Diquat dissolved in 20 mL of 0.9% sterile saline; 8 mg/kg BW Diquat dissolved in 20 mL of 0.9% sterile saline; and 10 mg/ kg BW Diquat dissolved in 20 mL of 0.9% sterile saline.

A Diquat dosage of 10 mg/kg BW was selected as the maximum dosage based on literature from mice (1), piglets (25), and sheep (26) demonstrating oxidative stress. Therefore, to the best of our knowledge, Diquat has not been evaluated in young ruminants (calves); thus, Diquat dosages of 6 and 8 mg/kg BW were chosen to minimize the risk of excessive oxidative stress and death. Diquat was purchased from Sigma Company (C_12_H_12_Br_2_N_2_ - H_2_O, molecular weight 362.06, product number: SIAL6385–62 - 2, purity ≥95%, presented as a yellow crystalline powder, with specifications of 250 mg/bottle).

All 12 Holstein calves were raised in 4 pens (1 pen/treatment) with free access to water and feed. Feed and refusals were recorded daily to calculate dry matter intake (DMI) for each pen using a digital scale (HCS3010D, Hua Chao, Shanghai, China). The calves received 4 kg of calf grower in 2 equal portions at 8 am and 3 pm daily, with orts collected before the morning feeding. A pelleted calf grower was purchased from Gansu Devotion Biotech Co., Ltd. (Lanzhou, China), and the ingredient composition and nutrient concentrations are provided in Table 1. The calculated DMI (kg/h/d) throughout the study was 3.17, 3.15, 2.67, and 2.54 kg/d for 0, 6, 8, and 10 mg/kg BW, respectively (not statistically analyzed). Although DMI is an important measurement, it was not a key parameter of this experiment.

**Table 1:** Ration ingredient composition and nutrient concentrations (% DM basis).

Ingredient	% of DM	Nutrient^a^	Concentration
Corn	44.47	DM	87.96
Soybean meal	24.50	CP	23.11
Wheat bran	8.00	EE	3.85
Cottonseed meal	4.80	CF	4.90
Puffed soybeans	4.00	Ash	7.80
Soy protein concentrate	2.00	NDF	12.34
Whey powder	4.00	ADF	5.79
Molasses	4.00	Ca	0.90
Calcium carbonate	1.40	P	0.60
Soybean oil	0.50		
Salt, white	0.80		
Dicalcium phosphate	0.30		
Magnesium oxide	0.20		
Premix^b^	1.00		
Flavor	0.03		
Total	100.00		

a DM, dry matter; CP, crude protein; EE, ether extract; CF, crude fiber; NDF, neutral detergent fiber; and ADF, Acid detergent fiber.

b Premix contains: Cu: 1000 mg/ kg, Fe: 5600 mg/ kg, Mn: 4200 mg/ kg, Zn: 9000 mg/ kg, I: 100 mg/ kg, Se: 40 mg/ kg, Co: 35 mg/ kg, Vit A 900 KIU/ kg, Vit D3 400 KIU/ kg, and Vit E 6500 IU/ kg.

Fecal scores were collected daily for each calf using a 5-point scale (23) (1 = stiff, 2 = pasty, 3 = regular, 4 = loose, 5 = watery), based on a modified version of the University of Wisconsin fecal scoring chart (32). Scores were determined through visual observation of calf defecation (Table 2). Fecal scores were compiled over 6-day periods, with scores greater than 3 classified as diarrhea. Fecal ratios were calculated as the number of diarrheic calves divided by the total number of calves.

**Table 2:** Fecal scoring criteria.

Grade	Phenotype	Morphological
1	Harder appearance	Normal shape
2	Soft and loose	Molding
3	Loose and watery	Shapeless
4	Watery, mucus	Shapeless
5	Watery, mucus, bloody	Shapeless

## Sample collections

### Growth performance

BW and body frame were measured daily before morning feeding on days 0, 6, 12, 18, and 24 using an electronic scale (Model XL3190-A12 + E, Yew Wah Weighing Systems Co., Ltd., Shanghai, China). Body frame measurements included body slope (from the anterior scapula to the posterior ipsilateral sciatic tubercle), body length (BL), withers height (WH), hip height (HH), heart girth (HG), cannon bone, and hip width (HW), which were measured using a Biltmore stick and a flexible ruler (Jiangsu Animal Husbandry Veterinary Equipment Manufacturing Co., Ltd., Jiangsu, China). Average daily gain (ADG) and body frame growth gains were calculated and recorded.

### Serum biochemical indices

Jugular blood samples were collected individually before morning feeding on days 0, 6, 12, 18, and 24. These samples were obtained via venipuncture into 10 mL Vacutainer serum separation tubes (Becton, Dickinson and Co., Franklin Lakes, NJ) using a 22-gauge, 3.18-cm needle. The samples were placed on ice and transported to the laboratory, where they were centrifuged (SN-LSC, Shanghai, China) at 2,000 x g for 20 min at room temperature. The serum was decanted and stored at −20°C for the determination of biochemical and antioxidant indexes. Jugular serum samples were thawed and analyzed for serum alanine transaminase (ALT), aspartate aminotransferase (AST), superoxide dismutase (SOD), glutathione peroxidase (GSH-PX), glutathione (GSH), catalase (CAT), total antioxidant capacity (T-AOC), malondialdehyde (MDA), cortisol (COR), and noradrenaline (NOR). These were measured by the Huaying Institute of Biotechnology (Beijing, China) using enzyme markers (Beijing Qian Biotechnology Co., Ltd.) and enzyme-linked immunosorbent assay kits (Wuhan Beiyin Biotechnology Co., Ltd., Wuhan, China).

### Fecal colony counting

Fresh fecal samples (5 mL) were collected from each calf before the morning feeding on days 0, 6, 12, 18, and 24 and stored in fecal sample collection tubes containing 20% glycerol at −20°C. Before enumerating the fecal colonies (*Escherichia coli*, *Staphylococcus aureus*, and *Salmonella-Shigella*), the fecal samples were thawed at room temperature. After thawing, 1 g of the fecal sample was taken, to which 5 mL of LB liquid medium was added and thoroughly mixed with shaking. Following the mixing, 1 mL of the mixture was pipetted into a conical flask using a sterile pipette gun, and 99 mL of saline was added. The flask was then sealed with sealing film and placed in a 37°C constant temperature shaker (180 r/min) for 24 h to allow the strain to recover. A pipette gun with a sterile tip was used to aspirate 1 mL of the resuscitated bacterial solution, and 99 mL of sterile saline was added and mixed vigorously before repeating the aforementioned procedure. The optimal dilution gradient for the culturing of *E. coli* (10^−6^), *S. aureus* (10^−2^), and *Salmonella-Shigella* (10^−6^) colonies was determined using the dilution plating method, and the *Petri* dishes were incubated at 37°C for 24 h post-injection, followed by counting. The CFUs were counted, and the counts were logarithmically transformed and expressed as log_10_ CFU/g.

### Statistical analysis

The data were organized using Excel (Microsoft Corp., Redmond, WA) and followed by checks for normality and outliers using the UNIVARIATE procedure of SAS (version 9.4, SAS Institute Inc., Cary, NC) before conducting any statistical analyses. Box and whisker plots and the Shapiro–Wilk Test were utilized to verify that the data were normally distributed (*p* > 0.15). Subsequently, all data were subjected to least squares analysis of variance (ANOVA) for an RCBD (30), comprising four treatments analyzed using the MIXED procedure of SAS, with a 6-day period considered as a repeated measures ANOVA. Statistical analyses were performed with individual calves as the experimental unit. The statistical model used was as follows:


$$Y_{ijk}=\mu +R_{i}+T_{j}+P_{k}+\left(T_{j}\;x\;P_{k}\right)+e_{ijk\text{,}}$$

where Yijk = dependent variable, μ = overall mean, R_i_ = replication, T_j_ = treatment; P_k_ = 6 d periods; T_j_ x P_k_ = interaction of treatment by 6 d period, and e_ijk_ = residual random error. Treatment effects were considered fixed, while replication was considered a random effect. The 6-day periods were considered repeated measurements over time with an autoregressive covariance structure. Although not explicitly coded in the model, the calf was considered a random factor. Polynomial contrasts were used to examine the linear, quadratic, and cubic effects of the treatments (i.e., 0, 6, 8, 10 mg/kg BW). Differences among treatments were examined using Duncan’s multiple range test when the F-test yielded significant results. All other data were analyzed using the same model described above, excluding the period and the interaction between the period and treatment. Statistical significance was defined as highly significant at a *p-value of* < 0.01, significant at a *p-value of* < 0.05, and trending at 0.05 < *p* ≤ 0.10. Pearson correlation analysis of serum oxidative stress indicators was conducted using SPSS 25.0 software, with highly significant correlations defined at *p* < 0.01 (two-sided) and significant correlations at a *p-value of* < 0.05 (bilateral).

## Results

### Diquat impacts on growth performance

A retrospective power and sample size analysis indicated that 12 animals across four treatments, with repeated measurements over time, would be sufficient to detect a 5% difference with a power greater than 85%, using a two-sided t-test for several key parameters. Since calves were injected individually, the only parameter that could not be measured on an individual basis was DMI. Although DMI is an important parameter, it was not the focus of this experiment. The critical study parameters collected on an individual animal basis included BW, BW gain, ADG, and blood measurements.

Calves were similar (*p* > 0.10) in BW across all treatments on day 0, as expected due to the blocking on BW (Table 3). The day proved significant (*p* < 0.05) for all parameters measured in this experiment but will not be discussed further. The interaction between treatment and day was significant (*p* < 0.01) for BW and ADG, with calves receiving increasing amounts of Diquat showing a cubic response, indicating a decrease in both BW and ADG at a diminishing rate. For some parameters, the inhibitory effects of Diquat on BW and ADG persisted for 18 days, after which the inhibitory effect on ADG was alleviated, while the reduction in BW continued through 24 days. The response data suggest that the optimal Diquat dosage for future oxidative stress experiments is 8 mg/kg BW.

**Table 3:** BW, ADG, and frame measurements of Holstein bull calves injected with Diquat at 0, 6, 8, and 10 mg/kg BW.

Measurement		Diquat, mg/kg BW					Trt^1^	TxD^2^	Contrast, *P* <		
	Day	0	6	8	10	SEM	*P* <	*P* <	Linear	Quadratic	Cubic
BW, kg	0	87.33	86.80	88.17	88.90	2.71	0.01	0.01	0.01	0.08	0.01
	6	97.67	96.33	89.50	90.50	2.71					
	12	99.92^a^	104.33^a^	92.83^b^	94.00^b^	2.71					
	18	108.50^a^	114.33^a^	102.00^b^	102.33^b^	2.71					
	24	127.33^a^	122.83^a^	111.83^b^	110.50^b^	2.71					
	Average	107.60^a^	104.93^a^	96.87^b^	97.25^b^	2.75					
ADG, kg/d	0–6	1.72^a^	1.59^a^	0.22^b^	0.23^b^	0.19	0.01	0.01	0.01	0.04	0.02
	7–12	1.81^a^	1.33^a^	0.56^b^	0.58^b^	0.19					
	13–18	1.44	1.67	1.53	1.39	0.19					
	19–24	1.69	1.42	1.64	1.36	0.19					
	0–24	1.67^a^	1.50^a^	0.99^b^	0.90^b^	0.10					
Body slope, cm	0	86.2	86.2	86.8	86.4	1.03	0.01	0.01	0.22	0.01	0.52
	6	89.1^b^	92.0^a^	91.8^ab^	89.8^b^	1.03					
	12	92.4^b^	95.2^a^	94.7^ab^	92.0^b^	1.03					
	18	94.6^b^	97.1^a^	95.8^ab^	93.2^b^	1.03					
	24	99.6^a^	97.6^b^	96.8^b^	96.1^b^	1.03					
Gain, cm	0–24	13.4^a^	11.4^b^	9.97^bc^	9.70^c^	0.71	0.01	-----	0.01	0.94	0.47
Body length, cm	0	82.8	83.5	83.0	83.6	1.13	0.01	0.01	0.01	0.18	0.09
	6	89.7^a^	85.8^bc^	87.8^ab^	83.0^c^	1.13					
	12	91.7^a^	89.4^b^	89.7^ab^	86.2^c^	1.13					
	18	94.5^a^	90.6^b^	91.5^b^	90.8^b^	1.13					
	24	95.6^a^	94.7^ab^	93.0^bc^	92.8^c^	1.13					
Gain, cm	0–24	12.8^a^	11.2^ab^	9.93^b^	9.23^b^	1.02	0.01	-----	0.01	0.678	0.64
Withers height, cm	0	94.50	94.4	94.0	93.3	0.85	0.03	0.72	0.01	0.29	0.69
	6	95.6	95.5	95.1	94.2	0.85					
	12	96.7^a^	96.1^ab^	96.0a^b^	94.7^b^	0.85					
	18	98.2	98.6	97.9	98.3	0.85					
	24	102.8^a^	101.5^b^	102.5^ab^	101.9^ab^	0.85					
Gain, cm	0–24	8.33	7.17	8.53	8.60	1.66	0.57	-----	0.77	0.29	0.63
Hip height, cm	0	101.3	100.5	99.8	99.6	0.92	0.62	0.86	0.21	0.72	0.69
	6	102.1	101.2	100.9	100.7	0.92					
	12	104.4	104.1	104.1	104.3	0.92					
	18	106.2	106.5	106.0	105.8	0.92					
	24	109.0	107.8	109.8	109.5	0.92					
Gain, cm	0–24	7.63^b^	7.33^ab^	9.93^a^	9.87^a^	1.13	0.03	-----	0.03	0.28	0.12
Heart girth, cm	0	105.0	105.2	105.4	105.4	0.58	0.01	0.16	0.01	0.02	0.16
	6	108.1^a^	106.3^b^	106.6^b^	106.2^b^	0.58					
	12	111.0^a^	109.6^b^	108.0^c^	107.0^d^	0.58					
	18	113.1^a^	111.9^b^	108.6^c^	108.5^c^	0.58					
	24	114.6^a^	113.6^b^	111.6^c^	109.8^d^	0.58					
Gain, cm	0–24	9.63^a^	8.37^ab^	6.20^bc^	4.40^c^	1.32	0.01	-----	0.01	0.06	0.74
Abdominal	0	116.3	116.6	116.9	116.8	0.99	0.01	0.01	0.01	0.01	0.01
Girth, cm	6	124.7^a^	119.9^b^	117.5^c^	118.5^c^	0.99					
	12	128.8^a^	125.6^b^	120.2^c^	122.4^c^	0.99					
	18	132.0^a^	132.5^a^	124.6^c^	123.6^c^	0.99					
	24	134.5^a^	133.4^a^	128.5^b^	125.8^c^	0.99					
Gain, cm	0–24	18.3^a^	16.8^a^	11.6^b^	8.97^c^	0.37	0.01	-----	0.01	0.01	0.04
Cannon bone, cm	0	12.5	12.5	12.6	12.5	0.23	0.16	0.80	0.11	0.10	0.54
	6	12.8	12.7	12.8	12.6	0.23					
	12	13.1	13.1	13.0	12.9	0.23					
	18	13.4	13.7	13.3	13.4	0.23					
	24	13.8	13.9	13.5	13.5	0.23					
Gain, cm	0–24	1.27	1.40	0.93	0.97	0.17	0.10	-----	0.13	0.18	0.14
Hip width, cm	0	17.4	17.5	17.3	17.6	0.59	0.83	1.00	0.93	0.79	0.44
	6	17.7	17.9	18.1	18.0	0.59					
	12	18.7	18.9	19.0	18.6	0.59					
	18	20.0	20.5	19.7	20.0	0.59					
	24	21.7	21.6	21.1	21.6	0.59					
Gain, cm	0–24	4.33	4.07	3.87	4.00	1.12	0.83	-----	0.69	0.92	0.82

1 Trt = Treatment.

2 TxD = Treatment by day interaction.

^a,b,c,d Means within the same row with different superscripts differ, p < 0.0.^

Calves injected with increasing amounts of Diquat demonstrated a treatment-by-day interaction for body slope (from the anterior border of the scapula to the posterior border of the ipsilateral sciatic tubercle), BL, and abdominal girth gains (*p* < 0.01). In contrast, treatment-by-day interactions were similar for weight height (WH), hip height (HH), heart girth (HG), cannon bone, and withers height (HW) gains (*p* > 0.10), as shown in Table 4. Calves treated with escalating doses of Diquat exhibited linear reductions in body slope, BL, HH, HG, and abdominal girth gains (*p* < 0.03), whereas WH, cannon bone, and HW gains remained similar (*p* > 0.10). Furthermore, calves injected with increasing Diquat dosages showed linear decreases in frame growth, noticeable as early as day 6 and persisting through day 24 for many frame growth parameters. The optimal Diquat dosage for influencing gains in frame growth parameters while elucidating oxidative stress could be as low as 6 mg/kg BW or lower.

**Table 4:** Serum indices of Holstein bull calves injected with Diquat at 0, 6, 8, and 10 mg/kg BW.

Measurement	Day	Diquat, mg/kg BW				SEM	Trt^1^	TxD^2^	Contrast, *P* <		
		0	6	8	10		*P* <	*P* <	Linear	Quadratic	Cubic
AST, U/L	0	75.0	74.7	71.4	72.7	2.59	0.01	0.01	0.01	0.12	0.17
	6	78.3^b^	81.5^b^	89.3^a^	89.8^a^	2.59					
	12	79.1^b^	87.2^a^	92.8^a^	91.6^a^	2.59					
	18	74.5^b^	78.9^b^	90.2^a^	94.5^a^	2.59					
	24	71.1^b^	77.7^ab^	81.0^ab^	86.5^a^	2.59					
ALT, U/L	0	18.8	18.6	18.4	18.3	1.02	0.01	0.01	0.01	0.01	0.04
	6	18.8	20.1	23.0	22.6	1.02					
	12	17.6^c^	18.7^c^	22.0^b^	25.6^a^	1.02					
	18	17.1^b^	19.1^b^	22.0^a^	24.4^a^	1.02					
	24	17.6^b^	18.0^b^	21.7^a^	22.8^a^	1.02					
SOD, U/L	0	89.4	87.8	88.1	89.1	3.49	0.01	0.02	0.01	0.02	0.18
	6	89.0^a^	81.6^ab^	83.1^ab^	77.1^b^	3.49					
	12	88.4^a^	86.1^a^	76.0^b^	75.7^b^	3.49					
	18	87.7^a^	98.4^a^	82.1^b^	75.0^c^	3.49					
	24	82.6^b^	86.2^b^	79.2^a^	77.1^a^	3.49					
GSH-Px, U/mL	0	353.1	355.1	351.2	349.9	11.73	0.01	0.06	0.01	0.02	0.39
	6	362.2^a^	348.0^a^	314.3^b^	307.1^b^	11.73					
	12	367.5^a^	336.0^b^	302.3^c^	288.5^c^	11.73					
	18	362.0^a^	347.0^ab^	328.7^bc^	304.1^c^	11.73					
	24	356.1	363.9	355.9	329.2	11.73					
GSH, umol/mL	0	8.05	8.80	7.87	8.62	0.62	0.01	0.20	0.01	0.12	0.37
	6	8.60^a^	7.68^a^	6.35^b^	6.16^b^	0.62					
	12	7.93^a^	7.79^ab^	6.37^bc^	6.12^c^	0.62					
	18	8.97^a^	8.49^a^	8.50^a^	6.89^b^	0.62					
	24	9.98^a^	8.25^ab^	7.85^b^	6.90^b^	0.62					
T-AOC, U/mL	0	8.85	7.92	7.42	7.60	0.55	0.01	0.87	0.01	0.67	1.00
	6	9.29^a^	7.61^b^	7.09^b^	7.12^b^	0.55					
	12	9.35^a^	7.87^ab^	6.99^b^	6.30^b^	0.55					
	18	9.78^a^	7.72^b^	7.43^b^	6.78^b^	0.55					
	24	9.78^a^	8.04^b^	7.93^b^	6.98^b^	0.55					
CAT, U/mL	0	53.9	51.4	51.7	53.7	2.97	0.01	0.01	0.01	0.64	0.34
	6	55.8	54.2	51.5	47.7	2.97					
	12	59.8^a^	42.7^b^	40.4^b^	38.6^b^	2.97					
	18	57.3^a^	50.3^b^	51.4^b^	41.5^c^	2.97					
	24	58.3^a^	54.7^a^	54.7^a^	48.9^b^	2.97					
MDA, nmol/mL	0	7.18	7.50	7.70	7.83	0.81	0.01	0.41	0.01	0.29	0.74
	6	7.86	8.76	8.81	9.07	0.81					
	12	6.79^d^	8.76^c^	10.2^b^	11.8^a^	0.81					
	18	7.24	7.56	8.66	9.24	0.81					
	24	6.37	7.19	7.86	8.22	0.81					
COR, ng/mL	0	38.4	39.6	38.6	39.4	0.89	0.01	0.04	0.01	0.36	0.62
	6	34.1^b^	37.2^ab^	40.4^a^	40.8^a^	0.89					
	12	35.3^c^	38.3^b^	38.4^b^	41.0^a^	0.89					
	18	35.0^b^	36.2^ab^	38.3^a^	38.8^a^	0.89					
	24	36.2^b^	36.4^b^	37.8^a^	37.6^a^	0.89					
NOR, pg./mL	0	325.8	326.3	328.5	328.3	11.92	0.01	0.01	0.01	0.01	0.01
	6	318.7^c^	356.5^b^	404.9^a^	417.8^a^	11.92					
	12	312.1^c^	368.3^b^	420.5^a^	431.2^a^	11.92					
	18	306.5^b^	305.0^b^	397.1^a^	405.5^a^	11.92					
	24	297.4^b^	285.4^b^	386.5^a^	408.2^a^	11.92					

1 Trt = Treatment.

2 TxD = Treatment by day interaction.

^a,b,c,d Means within the same row with different superscripts differ, p < 0.05.^

### Diquat impacts on fecal score and diarrhea ratios

Calves injected with increasing amounts of Diquat demonstrated a significant treatment-by-day interaction for fecal scores and Jaureguiberry’s scoring system (33) ratios (*p* < 0.01). Additionally, calves showed quadratic treatment responses (*p* < 0.02), with fecal scores and ratios increasing at a decreasing rate (see Table 5). By day 6 of the experiment, calves receiving Diquat to induce oxidative stress showed elevated fecal scores and ratios, which continued throughout the 24-day experimental period, with an optimal dosage of 8 mg/kg BW. The diarrhea rate was positively correlated with the Diquat dosage administered (*r* = 0.968, *p* = 0.032). Therefore, the optimal Diquat dosage for influencing fecal scores in the context of modeling oxidative stress is 6 mg/kg BW.

**Table 5:** Fecal scores and diarrhea ratio of Holstein bull calves injected with Diquat at 0, 6, 8, and 10 mg/kg BW.

Measurement	Day	Diquat, mg/kg BW				SEM	Trt^1^	TxD^2^	Contrast, *P* <		
		0	6	8	10		*P* <	*P* <	Linear	Quadratic	Cubic
Fecal score	0–6	1.82^c^	3.36^b^	4.35^a^	4.35^a^	0.18	0.01	0.01	0.01	0.02	0.46
	7–12	2.10^c^	2.44^c^	3.25^b^	4.06^a^	0.18					
	13–18	2.29^d^	2.74^c^	3.22^b^	4.21^a^	0.18					
	19–24	3.52^b^	4.60^a^	4.54^a^	4.66^a^	0.18					
	0–24	2.44^d^	3.29^c^	3.76^b^	4.32^a^	0.10					
Fecal ratios	0–6	8.33^c^	47.23^b^	69.45^a^	83.33^a^	10.51	0.01	0.10	0.01	0.01	0.31
	7–12	11.11^c^	55.56^b^	69.44^ab^	91.67^a^	10.51					
	13–18	19.44^c^	33.33^c^	66.67^b^	88.89^a^	10.51					
	19–24	30.56^b^	36.11^b^	63.89^a^	86.11^a^	10.51					
	0–24	17.36^d^	43.06^c^	67.36^b^	87.50^a^	5.79					

1 Trt = Treatment.

2 TxD = Treatment by day interaction.

^a,b,c,d Means within the same row with different superscripts differ, p < 0.05.^

### Diquat impacts on serum oxidative parameters

Calves injected with increasing amounts of Diquat showed significant increases (*p* < 0.04) in AST, ALT, SOD, CAT, COR, and NOR concentrations, while GSH-Px exhibited a tendency (*p* < 0.06). However, GSH, T-AOC, and MDA concentrations remained similar (*p* > 0.20) regarding the interaction of treatment by day, as shown in Table 4. Calves that received higher Diquat dosages indicated a cubic response for ALT and NOR concentrations, with ALT increasing at an accelerating rate, while SOD and GSH-PX concentrations decreased at a declining rate. Additionally, calves injected with more Diquat dosages demonstrated linear increases in AST, MDA, and COR, while GSH, T-AOC, and CAT concentrations decreased. Changes in serum parameters were observed by day 6 and as late as day 12 due to oxidative stress, with alterations persisting for many serum indices of oxidative stress up to day 24.

### Diquat correlation with blood indicators

As shown in Table 6, calves injected with Diquat demonstrated positive correlations (*p* < 0.05) with serum concentrations of AST, ALT, MDA, COR, and NOR, with correlation coefficients ranging from 0.53 to 0.62. The concentration of Diquat injection was negatively correlated (*p* < 0.05) with calf serum SOD, GSH-PX, GSH, T-AOC, and CAT concentrations, showing correlation coefficients between −0.50 and − 0.77, respectively. These correlations demonstrated that Diquat injection influenced serum markers related to oxidative stress in growing calves.

**Table 6:** Correlation of serum indices concentrations with Diquat injections at 0, 6, 8, and 10 mg/kg BW.

Parameter	AST	ALT	SOD	GSH-PX	GSH	T-AOC	CAT	MDA	COR	NOR
Concentration										
AST	1.00									
ALT	0.71**	1.00								
SOD	−0.49**	−0.63**	1.00							
GSH-PX	−0.71**	−0.64**	0.55**	1.00						
GSH	−0.62**	−0.55**	0.48**	0.47**	1.00					
T-AOC	−0.51**	−0.58**	0.39**	0.60**	0.45**	1.00				
CAT	−0.61**	−0.56**	0.42**	0.67**	0.53**	0.50**	1.00			
MDA	0.66**	0.52**	−0.50**	−0.55**	−0.49**	−0.44**	−0.54**	1.00		
COR	0.32*	0.49**	−0.39**	−0.49**	−0.44**	−0.60**	−0.43**	0.33**	1.00	
NOR	0.78**	0.85**	−0.72**	−0.47**	−0.64**	−0.58**	−0.62**	−0.64**	0.58**	1.00

^*^ Significantly correlated at the 0.01 level (bilateral). ^**^ Significantly correlated at the 0.05 level (two-sided).

### Diquat impacts on fecal pathogen concentrations

Calves injected with increasing amounts of Diquat demonstrated significant treatment-by-day interactions (*p* < 0.03) for fecal *Escherichia coli*, *Staphylococcus aureus*, and Salmonella-Shigella concentrations (Table 7). These calves exhibited a cubic response (*p* < 0.01), with fecal *Escherichia coli* concentrations initially increasing before decreasing. Increases in *Escherichia coli* concentrations appeared as early as day 6 and persisted throughout the 24 days. Calves injected with increasing Diquat dosages showed linear increases (*p* < 0.01) in fecal *Staphylococcus aureus* and Salmonella-Shigella concentrations, which were observed on days 6 and 18, respectively, lasting through day 24. The use of Diquat injections to elucidate oxidative stress indicates the development of oxidative stress associated with intestinal pathogen proliferation, supporting the use of Diquat in future studies to test feed additives that address oxidative stress issues at dosages of 8 mg/kg BW.

**Table 7:** Fecal pathogen concentrations in Holstein bull calves injected with Diquat at 0, 6, 8, and 10 mg/kg BW.

Measurement	Day	Diquat, mg/kg BW				SEM	Trt^1^	TxD^2^	Contrast, *P* <		
		0	6	8	10		*P* <	*P* <	Linear	Quadratic	Cubic
*Escherichia coli*, (10^−6^)	0	9.64	9.60	9.34	9.48	0.54	0.01	0.01	0.01	0.01	0.01
	6	11.4	11.4	13.3	12.9	0.54					
	12	13.5^bc^	13.0^c^	15.3^a^	14.9^ab^	0.54					
	18	12.8^b^	13.5^b^	14.6^ab^	16.3^a^	0.54					
	24	13.8^b^	12.9^b^	15.6^a^	15.7^a^	0.54					
*Staphylococcus aureus,* (10^−2^)	0	13.3	14.3	13.9	13.9	0.71	0.01	0.01	0.01	0.11	0.11
	6	14.8	15.1	16.0	16.1	0.71					
	12	16.3	15.6	16.8	17.2	0.71					
	18	14.1^b^	15.0^b^	17.1^a^	18.2^a^	0.71					
	24	14.0^b^	14.5^b^	15.2^ab^	16.18^a^	0.71					
*Salmonella-Shigella,* (10^−6^)	0	23.5	23.6	23.1	23.3	1.34	0.01	0.01	0.01	0.26	0.24
	6	24.1^b^	24.9^ab^	26.2^a^	25.9^ab^	1.34					
	12	27.2	28.4	28.3	28.2	1.34					
	18	26.7^c^	29.0^bc^	30.8^ab^	32.8^a^	1.34					
	24	27.4^b^	29.0^ab^	33.2^a^	34.6^a^	1.34					

1 Trt = Treatment.

2 TxD = Treatment by day interaction.

^a,b,c,d Means within the same row with different superscripts differ, P < 0.05.^

## Discussion

### Growth performance

This experiment demonstrates the development of oxidative stress in 70-day-old Holstein bull calves receiving an intraperitoneal Diquat injection. In this study, we administered different doses of Diquat via intraperitoneal injection to weaned calves to induce oxidative stress. Initially, vomiting and anorexia were observed in all treated calves following the injection. However, as the trial progressed, the feed intake of the treated calves improved, and all calves survived without mortality. These data support the hypothesis of using Diquat as an oxidant to induce oxidative stress over a period of 24 days. The induction of oxidative stress with increasing amounts of Diquat resulted in reductions in BW gains and ADG. Based on growth performance, the optimal dosage for future studies would be approximately 8 mg/kg BW.

The development of oxidative stress leads to the production of free radicals that exceed the body’s ability to metabolize them. Consequently, oxidative stress damages the intestinal biofilm, compromising the physiological function of intestinal cells and resulting in increasing digestive disorders, resulting in abnormal digestive secretions, intestinal damage, elevated intestinal mucosal permeability, and pathogenic infections, which manifest as decreased feed intake and reduced growth performance (34–36). These results are consistent with the research conducted by Yin et al. (20). In addition, these results indicated that the level of oxidative stress induced in calves was directly proportional to the concentration of Diquat injected.

Body frame measurements are an essential index for the development of calf growth during the post-weaning period (37). Diquat injections at all tested dosages in this experiment inhibited calf frame growth gains compared to calves injected with 0 mg/kg BW Diquat. The differences in heart girth and abdominal circumference gains could be related to the observed numerical reduction in DMI, which was affected by the degree of oxidative stress. The inhibitory effects of Diquat inducing oxidative stress persisted until day 24 of the experiment.

### Fecal scores and diarrhea ratios

The calf’s transition from a milk-only diet to solid feed is a very stressful period since the digestive system is not fully developed for nutrient absorption. The greater the stress levels during this transition, the more significant the oxidative stress becomes, which results in increased incidences of scours and a higher number of calves experiencing diarrhea. The oxidative stress during scours damages the tight junctions between intestinal cells (38, 39). The destruction of these junctions allows water and electrolytes (ions) to enter the intestinal lumen from the intestinal mucosa. Consequently, the resulting diarrhea diverts nutrients from growth performance to maintenance and the immune system, reducing growth, DMI, and FC, with potential mortality implications (40). In this experiment, calves receiving 6, 8, or 10 mg/kg Diquat developed diarrhea, and the fecal scores increased as the dosage of Diquat injected rose. Calves receiving the higher Diquat dosages were visually observed to have bloody stools during the last 6 days of the study period, indicating intestinal cellular damage and blood loss. An intraperitoneal injection of Diquat at 10 mg/kg BW as part of future oxidative stress experiments is not recommended, as calves showed visible signs of excessive stress.

### Serum biochemical indices

The metabolic disorders that accompany oxidative stress cause liver injury, leading to apoptosis and impairing hepatic and immune function (41). When the liver experiences reduced oxidative stress, nitrosylation of proteins, production of reactive oxygen species, and lipid peroxidation decrease as well, thus maintaining and restoring the integrity and function of the body’s intestinal barrier (42). Damage resulting from the intracellular release of ALT and AST evaluates blood ALT and AST concentrations, which decrease liver AST and ALT enzymatic activity. Increased blood AST and ALT concentrations indicate the degree of liver damage, thereby impairing liver function (43). Diquat injection raised blood AST and ALT concentrations by day 12, but these concentrations returned to baseline by day 24 of the experiment, similar to those of calves injected with 0 mg/kg BW. Yuan et al. reported that Diquat targets the liver, consistent with these data indicating liver damage in Diquat-injected calves (43, 44).

When animals are subjected to oxidative stress that produces free radicals, these radicals can be scavenged by antioxidant actives, including antioxidant enzymes and non-enzymatic antioxidant molecules, to protect the animal from oxidative damage. Non-enzymatic free radical scavengers include glutathione (GSH), selenium, several vitamins, and antioxidant enzymes such as SOD, GSH-Px, CAT, etc. (45). A common biomarker for free radicals is MDA, which is produced *in vivo* by the peroxidation of polyunsaturated fatty acids (46). COR plays a crucial role in regulating the association of immune cells with inflammation and in maintaining connective tissues. These data demonstrate that a single intraperitoneal Diquat injection in calves decreased serum concentrations of SOD, GSH-Px, GSH, CAT, and T-AOC while increasing concentrations of MDA and COR, which is consistent with the study by Yin et al. on piglets (20).

The neurotransmitter NOR can play a role in disease treatment, but prolonged high levels indicate that the body is under significant stress (47), which can lead to sustained increases in blood pressure and persistent hyperactivity, posing a threat to calf health and survival. In this experiment, NOR levels were significantly higher in the 8 and 10 mg/kg BW treatments; however, the responses are comparable between these two groups, indicating that at these concentrations, oxidative stress may be triggered in the body. Furthermore, these data demonstrate that serum SOD, GSH-Px, MDA, and COR concentrations are related to the severity of oxidative stress. The 6 mg/kg BW calves recovered by day 18, while calves receiving 8 and 10 mg/kg BW persisted until day 24.

### Fecal pathogenic bacteria

Pathogenic bacteria (*Escherichia coli*, *Staphylococcus aureus*, and *Salmonella*) are among the primary causes of calf scours (48)^,^ which can result in high morbidity and mortality, significantly impacting calf performance and economics (49). These data demonstrate that increasing the Diquat injection dosage intensifies both the severity and duration of stress, resulting in elevated concentrations of fecal pathogenic bacteria that affect the health status of calves, especially during the later stages of the experiment. An increase in Diquat dosage correlates with a rise in pathogenic bacteria abundance, likely due to the calf’s impaired immunocompetence caused by oxidative damage to the intestinal barrier, which leads to reduced DMI, digestive metabolism, and nutrient absorption. Calves depend on proteins, minerals, carbohydrates, and other nutrients for immune regulation (50). If a calf cannot consume sufficient nutrients to support its immune system and maintain intestinal nutrient absorption, pathogenic bacteria will proliferate, leading to a decline in immunocompetence and an increased risk of infection (51).

## Conclusion

This study demonstrated that a single intraperitoneal injection of three Diquat concentrations (6, 8, and 10 mg/kg BW) can induce oxidative stress in calves. The trial confirmed that increasing Diquat dosages increased oxidative stress severity, as evidenced by reduced growth performance, body frame gains, altered serum oxidative stress indices, and increased fecal scores and pathogen concentrations. However, the severity and duration of oxidative stress varied with the dosage of Diquat injected. Based on these data, the recommended Diquat dosage for future studies would be 8 mg/kg BW, as it may persist for 18 to 24 days, depending on the parameter, while 10 mg/kg BW is not recommended from an animal welfare perspective due to visible signs of excessive stress and the appearance of bloody stools.

## Data Availability

The original contributions presented in the study are included in the article/supplementary material, further inquiries can be directed to the corresponding author.

## References

[1] Fu Y Cheng WH Ross DA Xg L. Cellular glutathione peroxidase protects mice against lethal oxidative stress induced by various doses of diquat. Proc Soc Exp Biol Med. (1999) 222:164–9. doi: 10.1046/j.1525-1373.1999.d01-127.x, PMID: 10564541

[2] Lyu Y Bai L Qin C. Long noncoding RNAs in neurodevelopment and Parkinson's disease. Animal Model Exp Med. (2019) 2:239–51. doi: 10.1002/ame2.12093, PMID: 31942556 PMC6930994

[3] de Souza TO Kuczynski da Rocha M Gil Sessim A. Weaning at 30, 75 and 180 days: comparison between immune responses of beef calves. Res Vet Sci. (2021) 138:53–61. doi: 10.1016/j.rvsc.2021.06.002, PMID: 34111714

[4] Kim MH Yang JY Upadhaya SD Lee HJ Yun CH Ha JK. The stress of weaning influences serum levels of acute-phase proteins, iron-binding proteins, inflammatory cytokines, cortisol, and leukocyte subsets in Holstein calves. J Vet Sci. (2011) 12:151–7. doi: 10.4142/jvs.2011.12.2.151, PMID: 21586874 PMC3104169

[5] Jasper J Budzynska M Weary DM. Weaning distress in dairy calves: acute behavioural responses by limit-fed calves. Appl Anim Behav Sci. (2008) 110:136–43. doi: 10.1016/j.applanim.2007.03.017

[6] Lightfoot TJ Skibola CF Smith AG Forrest MS Adamson PJ Morgan GJ. Polymorphisms in the oxidative stress genes, superoxide dismutase, glutathione peroxidase and catalase and risk of non-Hodgkin's lymphoma. Haematologica. (2006) 91:1222–7. PMID: 16956821

[7] Gallagher EP Buetler TM Stapleton PL Wang C Stahl DL Eaton DL. The effects of diquat and ciprofibrate on mRNA expression and catalytic activities of hepatic xenobiotic metabolizing and antioxidant enzymes in rat liver. Toxicol Appl Pharmacol. (1995) 134:81–91. doi: 10.1006/taap.1995.1171, PMID: 7676460

[8] Schallreuter KU Gibbons NC Zothner C Abou Elloof MM Wood JM. Hydrogen peroxide-mediated oxidative stress disrupts calcium binding on calmodulin: more evidence for oxidative stress in vitiligo. Biochem Biophys Res Commun. (2007) 360:70–5. doi: 10.1016/j.bbrc.2007.05.218, PMID: 17592724

[9] Yuan SB Chen DW Zhang KY Yu B. Effects of oxidative stress on growth performance, nutrient digestibilities and activities of antioxidative enzymes of weanling pigs. Asian Australas J Anim Sci. (2007) 20:1600–5. doi: 10.5713/ajas.2007.1600

[10] Wijtten PJ van der Meulen J Verstegen MW. Intestinal barrier function and absorption in pigs after weaning: a review. Br J Nutr. (2011) 105:967–81. doi: 10.1017/S0007114510005660, PMID: 21303573

[11] Henderson P van Limbergen JE Schwarze J Wilson DC. Function of the intestinal epithelium and its dysregulation in inflammatory bowel disease. Inflamm Bowel Dis. (2011) 17:382–95. doi: 10.1002/ibd.21379, PMID: 20645321

[12] Gallagher EP Sheehy KM. Effects of phenytoin on glutathione status and oxidative stress biomarker gene mRNA levels in cultured precision human liver slices. Toxicol Sci. (2001) 59:118–26. doi: 10.1093/toxsci/59.1.118, PMID: 11134551

[13] Zhang C Walker LM Hinson JA Mayeux PR. Oxidant stress in rat liver after lipopolysaccharide administration: effect of inducible nitric-oxide synthase inhibition. J Pharmacol Exp Ther. (2000) 293:968–72. doi: 10.1016/S0022-3565(24)39321-8, PMID: 10869399

[14] Rikans LE Cai Y. Age-associated enhancement of diquat-induced lipid peroxidation and cytotoxicity in isolated rat hepatocytes. J Pharmacol Exp Ther. (1992) 262:271–8. doi: 10.1016/S0022-3565(25)10747-7, PMID: 1320687

[15] Hurst J Kuehn S Jashari A Tsai T Bartz-Schmidt KU Schnichels S. A novel porcine ex vivo retina culture model for oxidative stress induced by H₂O₂. Altern Lab Anim. (2017) 45:11–25. doi: 10.1177/026119291704500105, PMID: 28409994

[16] Wang L Chen Q Zhuang S Wen Y Cheng W Zeng Z. Effect of Anoectochilus roxburghii flavonoids extract on H_2_O_2_- induced oxidative stress in LO2 cells and D-gal induced aging mice model. J Ethnopharmacol. (2020) 254:112670. doi: 10.1016/j.jep.2020.112670, PMID: 32135242

[17] Hwang DF Hour JL Cheng HM. Effect of taurine on toxicity of oxidized fish oil in rats. Food Chem Toxicol. (2000) 38:585–91. doi: 10.1016/s0278-6915(00)00052-1, PMID: 10942319

[18] Zhang J Wang Z Shi Y Xia L Hu Y Zhong L. Protective effects of chlorogenic acid on growth, intestinal inflammation, hepatic antioxidant capacity, muscle development and skin color in channel catfish *Ictalurus punctatus* fed an oxidized fish oil diet. Fish Shellfish Immunol. (2023) 134:108511. doi: 10.1016/j.fsi.2022.108511, PMID: 36599381

[19] Brandsch C Nass N Eder K. A thermally oxidized dietary oil does not lower the activities of lipogenic enzymes in mammary glands of lactating rats but reduces the milk triglyceride concentration. J Nutr. (2004) 134:631–6. doi: 10.1093/jn/134.3.631, PMID: 14988459

[20] Yin J Liu M Ren W Duan J Yang G Zhao Y. Effects of dietary supplementation with glutamate and aspartate on diquat-induced oxidative stress in piglets. PLoS One. (2015) 10:e0122893. doi: 10.1371/journal.pone.0122893, PMID: 25875335 PMC4398417

[21] Zhang X Wang S Wu Y Liu X Wang J Han D. Ellagic acid alleviates Diquat-induced jejunum oxidative stress in C57BL/6 mice through activating Nrf2 mediated signaling pathway. Nutrients. (2022) 14:1103. doi: 10.3390/nu14051103, PMID: 35268077 PMC8912502

[22] Yang L Cheng J Xu D Zhang Z Hua R Chen H. Melatonin ameliorates Diquat-induced testicular toxicity via reducing oxidative stress, inhibiting apoptosis, and maintaining the integrity of blood-testis barrier in mice. Toxics. (2023) 11:160. doi: 10.3390/toxics11020160, PMID: 36851035 PMC9958747

[23] Zha P Wei L Liu W Chen Y Zhou Y. Effects of dietary supplementation with chlorogenic acid on growth performance, antioxidant capacity, and hepatic inflammation in broiler chickens subjected to diquat-induced oxidative stress. Poult Sci. (2023) 102:102479. doi: 10.1016/j.psj.2023.102479, PMID: 36669355 PMC9871335

[24] Wu F Yang X Wang F Liu Y Han S Liu S. Dietary curcumin supplementation alleviates diquat-induced oxidative stress in the liver of broilers. Poult Sci. (2023) 102:103132. doi: 10.1016/j.psj.2023.103132, PMID: 37826902 PMC10571021

[25] Lv M Yu B Mao XB Zheng P He J Chen DW. Responses of growth performance and tryptophan metabolism to oxidative stress induced by diquat in weaned pigs. Animal. (2012) 6:928–34. doi: 10.1017/S1751731111002382 22558963

[26] Zhao YB. The relieving effects and mechanism of allium mongolicum regeland its flavonoids on small tail Han sheep in stress condition induced by diquat. Hohhot: Inner-Mongolia Agricultural University (2019).

[27] Liu L Chen D Yu B Luo Y Huang Z Zheng P. Influences of selenium-enriched yeast on growth performance, immune function, and antioxidant capacity in weaned pigs exposure to oxidative stress. Biomed Res Int. (2021) 2021:5533210. doi: 10.1155/2021/5533210, PMID: 33855070 PMC8019624

[28] ZhengMing H. Guidelines for the management and use of laboratory animals. 1st. ed. Beijing: Science Press (2016).

[29] ADSA-ASAS-PSA. Guide for the care and use of agricultural animals in research and teaching teaching In: Tucker CB (editor). American dairy science association, American Society of Animal Science, and poultry science association (2020)

[30] Steel RGD Torrie JH. Principles and procedures of statistics. 2nd ed. New York: McGraw-Hill BookCo (1980).

[31] Saif LJ Smith KL. Enteric viral infections of calves and passive immunity. J Dairy Sci. (1985) 68:206–28. doi: 10.3168/jds.S0022-0302(85)80813-4, PMID: 2984270 PMC7130765

[32] McGuirk S. (2013). Calf health scoring chart. Calf health scoring chart. University of Wisconsin, School of Veterinary Medicine. Available at: https://www.vetmed.wisc.edu/dms/fapm/fapmtools/8calf/calf_health_scoring_chart.pdf (Accessed September 1, 2015).

[33] Jaureguiberry M Rearte R Marconi MJ Giuliodori MJ Madoz LV Pinedo FA. A simplified scoring system for the diagnosis of diarrhea and respiratory diseases in dairy calves. Can Vet J. (2023) 64:553–7. PMID: 37265806 PMC10204880

[34] Tian J Jiang Q Bao X Yang F Li Y Sun H. Plant-derived squalene supplementation improves growth performance and alleviates acute oxidative stress-induced growth retardation and intestinal damage in piglets. Anim Nutr. (2023) 15:386–98. doi: 10.1016/j.aninu.2023.09.001, PMID: 38058564 PMC10695848

[35] Zha A Yan J Li J Wang J Qi M Liao P. Melatonin increased antioxidant capacity to ameliorate growth retardation and intestinal epithelial barrier dysfunction in diquat-challenged piglets. J Sci Food Agric. (2024) 104:2262–71. doi: 10.1002/jsfa.13114, PMID: 37947497

[36] Liang C Ren Y Tian G He J Zheng P Mao X. Dietary glutathione supplementation attenuates oxidative stress and improves intestinal barrier in diquat-treated weaned piglets. Arch Anim Nutr. (2023) 77:141–54. doi: 10.1080/1745039X.2023.2199806, PMID: 37133420

[37] Liu T Chen H Bai Y Wu J Cheng S He B. Calf starter containing a blend of essential oils and prebiotics affects the growth performance of Holstein calves. J Dairy Sci. (2020) 103:2315–23. doi: 10.3168/jds.2019-16647, PMID: 31980222

[38] Hulbert LE Moisá SJ. Stress, immunity, and the management of calves. J Dairy Sci. (2016) 99:3199–216. doi: 10.3168/jds.2015-10198, PMID: 26805993

[39] Özkaya S Kumbul BS Erbaş S Mutlucan M Altinay R Demirel E. Effects of garlic powder-supplemented milk on growth and health indicators in Holstein calves. Anim Biotechnol. (2023) 34:1–8. doi: 10.1080/10495398.2023.2230475 37409541 PMC13353482

[40] Urie NJ Lombard JE Shivley CB Kopral CA Adams AE Earleywine TJ. Preweaned heifer management on US dairy operations: part V. Factors associated with morbidity and mortality in preweaned dairy heifer calves. J Dairy Sci. (2018) 101:9229–44. doi: 10.3168/jds.2017-14019, PMID: 29935825 PMC7094390

[41] Liu H Huang Y Huang X Li MH Chen D Geng Y. *Eucommia ulmoides* Oliver enhances the antioxidant capacity and protects *Micropterus salmoides* from liver damage and immune function impairment caused by a high starch diet. J Funct Foods. (2023) 101:105424. doi: 10.1016/j.jff.2023.105424

[42] Pirozzi C Lama A Simeoli R Paciello O Pagano TB Mollica MP. Hydroxytyrosol prevents metabolic impairment reducing hepatic inflammation and restoring duodenal integrity in a rat model of NAFLD. J Nutr Biochem. (2016) 30:108–15. doi: 10.1016/j.jnutbio.2015.12.004, PMID: 27012627

[43] Xiong X Ren Y Cui Y Li R Wang C Zhang Y. Obeticholic acid protects mice against lipopolysaccharide-induced liver injury and inflammation. Biomed Pharmacother. (2017) 96:1292–8. doi: 10.1016/j.biopha.2017.11.083, PMID: 29174575

[44] Yuan D Hussain T Tan B Liu Y Ji P Yin Y. The evaluation of antioxidant and anti-inflammatory effects of *Eucommia ulmoides* flavones using Diquat-challenged piglet models. Oxidative Med Cell Longev. (2017) 2017:8140962. doi: 10.1155/2017/8140962, PMID: 28894511 PMC5574320

[45] Cadenas E Davies KJ. Mitochondrial free radical generation, oxidative stress, and aging. Free Radic Biol Med. (2000) 29:222–30. doi: 10.1016/s0891-5849(00)00317-8 11035250

[46] Sehirli O Tozan A Omurtag GZ Cetinel S Contuk G Gedik N. Protective effect of resveratrol against naphthalene-induced oxidative stress in mice. Ecotoxicol Environ Saf. (2008) 71:301–8. doi: 10.1016/j.ecoenv.2007.08.023, PMID: 18261796

[47] Gotovina J Pranger CL Jensen AN Wagner S Kothgassner OD Mothes-Luksch N. Elevated oxytocin and noradrenaline indicate higher stress levels in allergic rhinitis patients: implications for the skin prick diagnosis in a pilot study. PLoS One. (2018) 13:e0196879. doi: 10.1371/journal.pone.0196879, PMID: 29813071 PMC5973608

[48] Kim HS Whon TW Sung H Jeong YS Jung ES Shin NR. Longitudinal evaluation of fecal microbiota transplantation for ameliorating calf diarrhea and improving growth performance. Nat Commun. (2021) 12:389. doi: 10.1038/s41467-020-20389-5, PMID: 33420064 PMC7794225

[49] Cho YI Yoon KJ. An overview of calf diarrhea - infectious etiology, diagnosis, and intervention. J Vet Sci. (2014) 15:1–17. doi: 10.4142/jvs.2014.15.1.1 24378583 PMC3973752

[50] Gasmi A Piscopo S Menzel A Noor S. A review on metabolic paradoxes and their impact on metabolism. Arch Razi Inst. (2022) 77:929–41. doi: 10.22092/ARI.2021.356277.1815, PMID: 36618306 PMC9759232

[51] Tourkochristou E Triantos C Mouzaki A. The influence of nutritional factors on immunological outcomes. Front Immunol. (2021) 12:665968. doi: 10.3389/fimmu.2021.665968, PMID: 34135894 PMC8201077

